# Hierarchical decision model for *in vitro* bilirubin content prediction from absorption spectrum of whole blood

**DOI:** 10.1117/1.JBO.28.6.067001

**Published:** 2023-06-29

**Authors:** Swati Mishra, Harshawardhan Wanare

**Affiliations:** a Indian Institute of Technology, Department of Biological Sciences and Bioengineering, Kanpur, Uttar Pradesh, India; b Indian Institute of Technology, Centre of Lasers and Photonics, Kanpur, Uttar Pradesh, India; c Indian Institute of Technology, Department of Physics, Kanpur, Uttar Pradesh, India

**Keywords:** bilirubin, whole blood, absorption spectrum, correlation, hierarchical decision model, prediction accuracy

## Abstract

**Significance:**

Bilirubin forms by the breakdown of heme proteins in the liver, but a newborn’s sluggish liver can lead to elevated serum bilirubin levels that cross the blood–brain barrier and result in kernicterus. Earlier studies have used the 400 to 500 nm optical wavelength range to characterize the bilirubin content. There is not a universally established correlation among other wavelengths and the amount of bilirubin in clinical whole blood samples.

**Aim:**

We demonstrated that the amount of bilirubin could be quantified with ∼82% accuracy in a label-free, self-referenced manner using only a few wavelengths, viz. 468, 492, 500, 560, 605, 645, 660, and 675 nm, wherein band-averaged absorption measurements are used.

**Approach:**

We addressed the above problem by conducting a preliminary study containing 50 neonates through an absorption spectrum measurement of whole blood in 3 to 5  μl samples from the neonates. We constructed a hierarchical decision method that first grossly divides the 30 neonates of the training set into <10  mg/dl and ≥10  mg/dl bilirubin level cohorts. A subsequent boundary condition further divides the ≥10  mg/dl group into two >15  mg/dl and ≤15  mg/dl bilirubin level cohorts. A finer measure later predicted the bilirubin content of each of these groups as low (<10  mg/dl), medium (10 to 15  mg/dl), and high (>15  mg/dl).

**Results:**

Using this hierarchical decision model statistical approach, we quantified the amount of bilirubin in the 20 testing set samples with 82% accuracy.

**Conclusions:**

We formulated a biostatistical model in which we automated the spectrometric determination of total bilirubin in the whole blood for patients of neonatal hyperbilirubinemia.

## 1 Introduction

Unconjugated bilirubin is formed as a result of heme catabolism. Due to its lipophilic nature, it binds to the albumin proteins and is transported to the liver. The uridine diphosphate-glucuronosyltransferase enzyme adds glucuronic acid groups to increase the solubility of this unconjugated bilirubin, thereby enhancing its elimination from the body. This conjugation process also does not allow the bilirubin biomolecule to cross the blood–brain barrier. In neonates, an inefficient uridine diphosphate-glucuronosyltransferase enzyme in their sluggish liver can cause unconjugated hyperbilirubinemia, leading the bilirubin to diffuse across the blood–brain barrier and result in kernicterus.^1^ Neonatal hyperbilirubinemia or jaundice affects 60% of newborns and 80% of preterm infants worldwide, making its screening, diagnostics, and therapy of imminent importance.^2^ Phototherapy is provided to those neonates that suffer from this pathophysiological condition. Before and during the phototherapy, it is imperative to sample the bilirubin concentration in the blood or serum of the subject to determine the duration and the effectiveness of the therapy. Many transcutaneous bilirubinometers that are easier to operate than a more elaborate spectrometric determination of bilirubin are available in the market. These are point-of-care, non-invasive, stress-free, and infection-free procedures for the newborn. They use the real-time multiwavelength reflectance method to determine the spectral property of the skin concerning the different levels of bilirubin in the blood. However, the results from these devices may not be ubiquitously acceptable because they are dependent on the heme content beneath the skin, the thickness of the dermal and epidermal layers, melanin, and fat content. They are also limited in use as they can effectively diagnose bilirubin levels <12 to 13  mg/dl, whereas in cases in which the levels may rise to 15 to 20  mg/dl, the measures from transcutaneous bilirubinometers are not considered reliable.^3^ Clinical neonatologists favor the conventional invasive serum biochemical tests more than transcutaneous bilirubinometery for effective bilirubin measurement. These conventional serum tests use certain reagents to produce colored products that are analyzed with the help of a spectrometer, and the serum bilirubin is correspondingly estimated. The main drawback of the conventional method is a larger amount of blood is required (∼5  ml).^4^ Here, we specifically use only microliters (two to five drops) of blood.

The reason for the inaccurate determination of bilirubin levels in the blood or serum of neonates resides in photoisomerizing its dynamic nature in which the bilirubin isomers complicate the situation. Bilirubin molecules absorb light to undergo rapid photoisomerization. Most of these photoisomers of bilirubin have not been chemically characterized to date. Due to their instability, they undergo reversible photoisomerization iteratively to form other intermediates. This photoisomerization process in the solution leads to the formation of many chemical species that interfere with the native bilirubin reaction, making it difficult to estimate the bilirubin concentration in serum accurately.^5^^–^^7^ Another reason is that the procedures mentioned in the literature concerning determining total bilirubin are not direct spectrometric methods, that is, the calibration has been carried out by adding diazo reagents in bulk or using spiked bilirubin samples.^8^ The presence of other chromophores in blood, such as oxyhemoglobin, hemoglobin, and biliverdin, is also responsible for erratic results.^9^ Pediatricians, therefore, feel the need for a direct screening method with a fast turnaround. Earlier groups have spectrometrically analyzed this contentious biomolecule and estimated its absorbance at various wavelengths between 400 and 500 nm (417, 456, 460, 471, 490 nm), varying with the site from which the sample was collected.^5^^,^^9^^–^^11^

We present a preliminary study of 50 neonates (maximum age being 1 week after birth) suspected of suffering from neonatal hyperbilirubinemia. Initially, we categorized the 30 newborns of the training set into two groups of <10  mg/dl (low) and 10 to 20  mg/dl based on their characteristic absorption spectra. Thereafter, we estimated another measure to divide the 10 to 20  mg/dl group into ≤15  mg/dl (medium) and >15  mg/dl (high) bilirubin concentration cohorts. According to the NICU guidelines, valid for 2022, provided by health institutions such as NHS and other pediatric hospitals worldwide, the management of neonatal jaundice is performed differently for neonates with bilirubin concentrations in a particular range. The frequency of monitoring serum bilirubin and the duration of phototherapy sessions are decided accordingly. Most of these guidelines use cutoffs of <10, 10 to 15, 15 to 20, and >20  mg/dl. These numbers are taken into account by clinical neonatologists while treating a neonate with jaundice. Hence, our cutoffs are based on these ranges.^12^^,^^13^ We further refined the measurement using the absorption spectra in the wavelength range peculiar to bilirubin to find the correlation between the actual bilirubin values and the absorption intensity of whole blood. The model’s reliability was checked by accurately predicting the bilirubin concentration on the remaining 20 unknown whole blood samples from the laboratory. Earlier research has not established a conclusive correlation with the amount of bilirubin using the absorption spectra in clinical whole blood samples of neonates to the best of our knowledge. In contrast to convention, we use only a few microliters of blood in a microcapillary without adding any reagents to the whole blood sample.

The remainder of the paper is organized as follows: Sec. 2 presents the materials and methods used in the proposed work. In Sec. 3, we experimentally demonstrate the proposed hierarchical decision model from the training set samples and later prove its efficacy in terms of prediction accuracy of the bilirubin content in the test samples. Finally, Sec. 4 concludes the paper with a discussion on the limitations and future scope of the work in the healthcare domain.

## 2 Materials and Methods

**Fig. 1:**
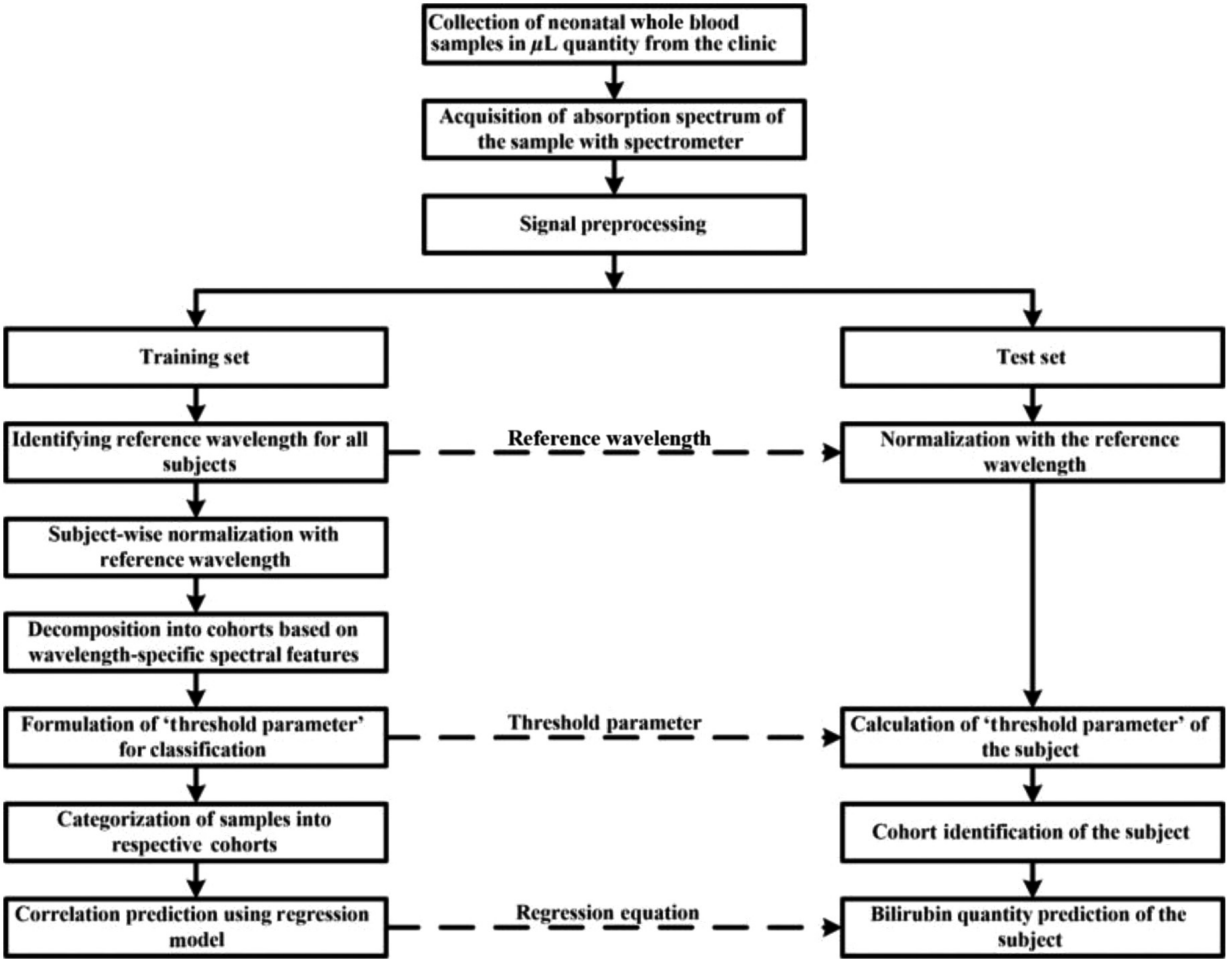
Block diagram of the proposed approach.

Our proposed modality is a potent screening method that works effectively with whole blood samples in real time while not being computationally intensive. This technique involves a finite set of band limited absorption spectrum signals accompanied by a decision tree-like analysis algorithm that involves computing only a few arithmetic operations. We are extending the preliminary study of lab experiments of spiked bilirubin concentrations to the whole blood samples.^14^ A block diagram for the proposed approach is elaborated in Fig. 1.

Clinical whole blood samples from 50 neonates suspected of suffering from neonatal hyperbilirubinemia were collected in ethylenediamine tetraacetic acid coated vials wrapped in aluminum foil to avoid exposure to ambient light.

### 2.1 Signal Acquisition

**Fig. 2:**
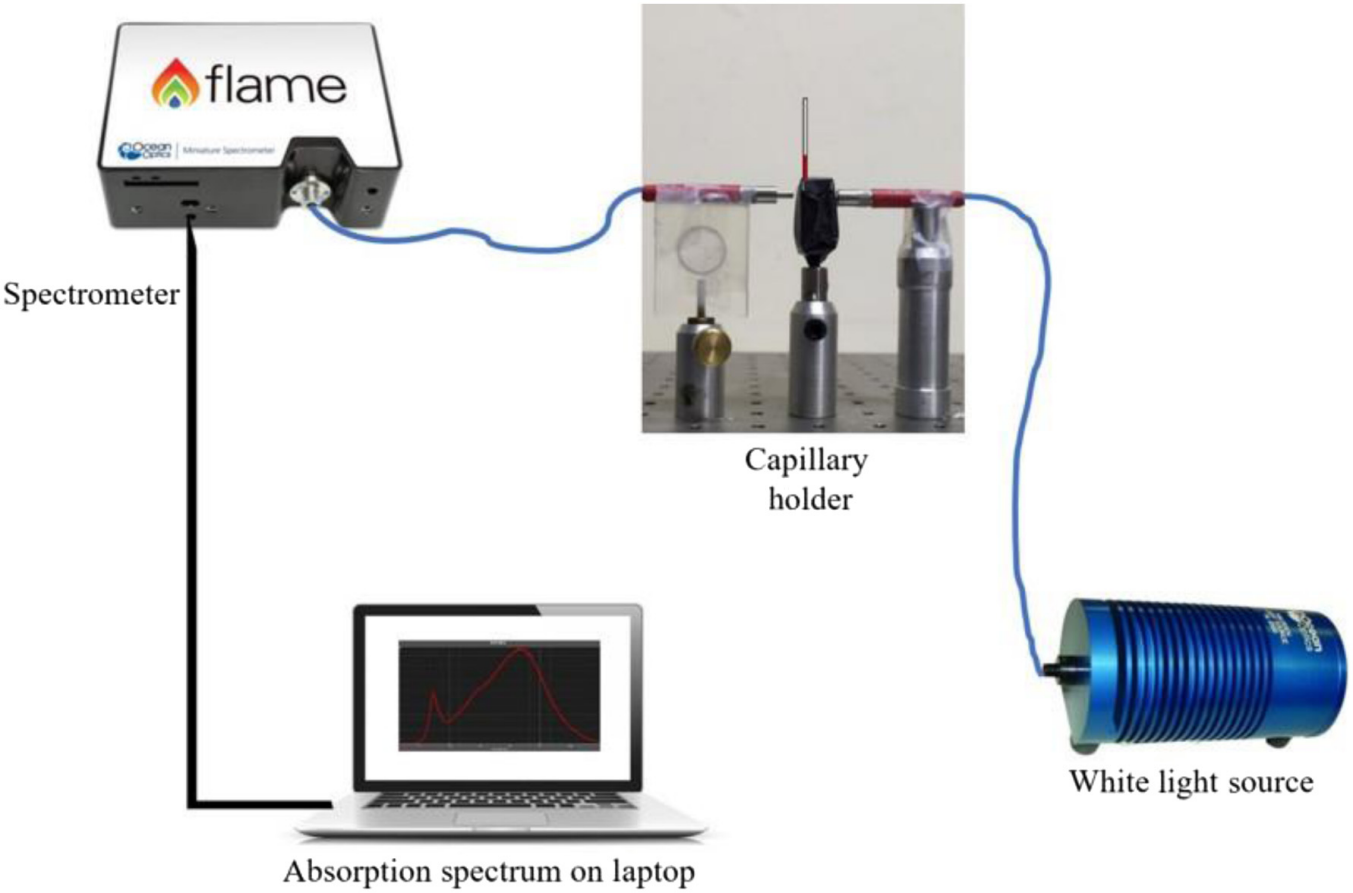
Schematic representation of the setup for recording the absorption spectrum of neonatal whole blood samples loaded in the capillary. The capillary holder is illuminated with the white light source and with the help of the spectrometer; the intensity with respect to wavelength is displayed on the computer.

The blood sample was loaded in an ordinary glass microcapillary (about a few μL), and the sample was illuminated with white light from an Ocean Optics HL-2000-HP tungsten halogen lamp. The broad spectrum of the white light source allows us to explore the distinguishing features of the absorption spectra among the cohorts that may potentially be used to correlate with the total bilirubin levels. The transmitted light from the whole blood sample in the glass microcapillary is collected as a signal through a fiber-coupled Ocean Optics FLAME-T-VIS-NIR-ES spectrometer. This spectrometer displays an absorption spectrum over a 340 to 1060 nm wavelength range with an optical resolution of 0.2 nm. The errors in alignment of the glass microcapillary are eliminated during the optical measurements using an in-house developed holder, a bilirubinometer, made of a black acrylate polymer, which stably holds the glass capillary containing the sample in position. The microcapillary groove in the bilirubinometer device is 2 mm, and a cylindrical glass capillary of the same outer diameter and 0.5 mm glass thickness was used for measurements. The optical fibers from the light source and the spectrometer are aligned with the microcapillary through a pinhole, and a fresh microcapillary can be conveniently replaced without disturbing the optical alignment. The optical spectra were obtained with the OceanView Software Suite^15^ running on a laptop. Figure 2 shows the schematics of the setup.

### 2.2 Signal Preprocessing

The raw spectrometric data are normalized using the statistical software Origin 2021b.^16^ The intensity counts against each wavelength are normalized between 0 and 1 for each participant to compare the graphs effectively using Eq. (1):

$$I^{\prime }(\lambda )=\frac{I(\lambda )-I_{\min}}{I_{\max}-I_{\min}},$$ (1)

where $I^{\prime }(\lambda )$ denotes the normalized intensity and I(λ) is the recorded spectrometric intensity count at that specific wavelength λ. $I_{\min}$ and $I_{\max}$ are the minimum and maximum intensity recorded over the entire wavelength range for that particular sample, respectively.

### 2.3 Proposed Hierarchical Decision Tree Model

**Fig. 3:**
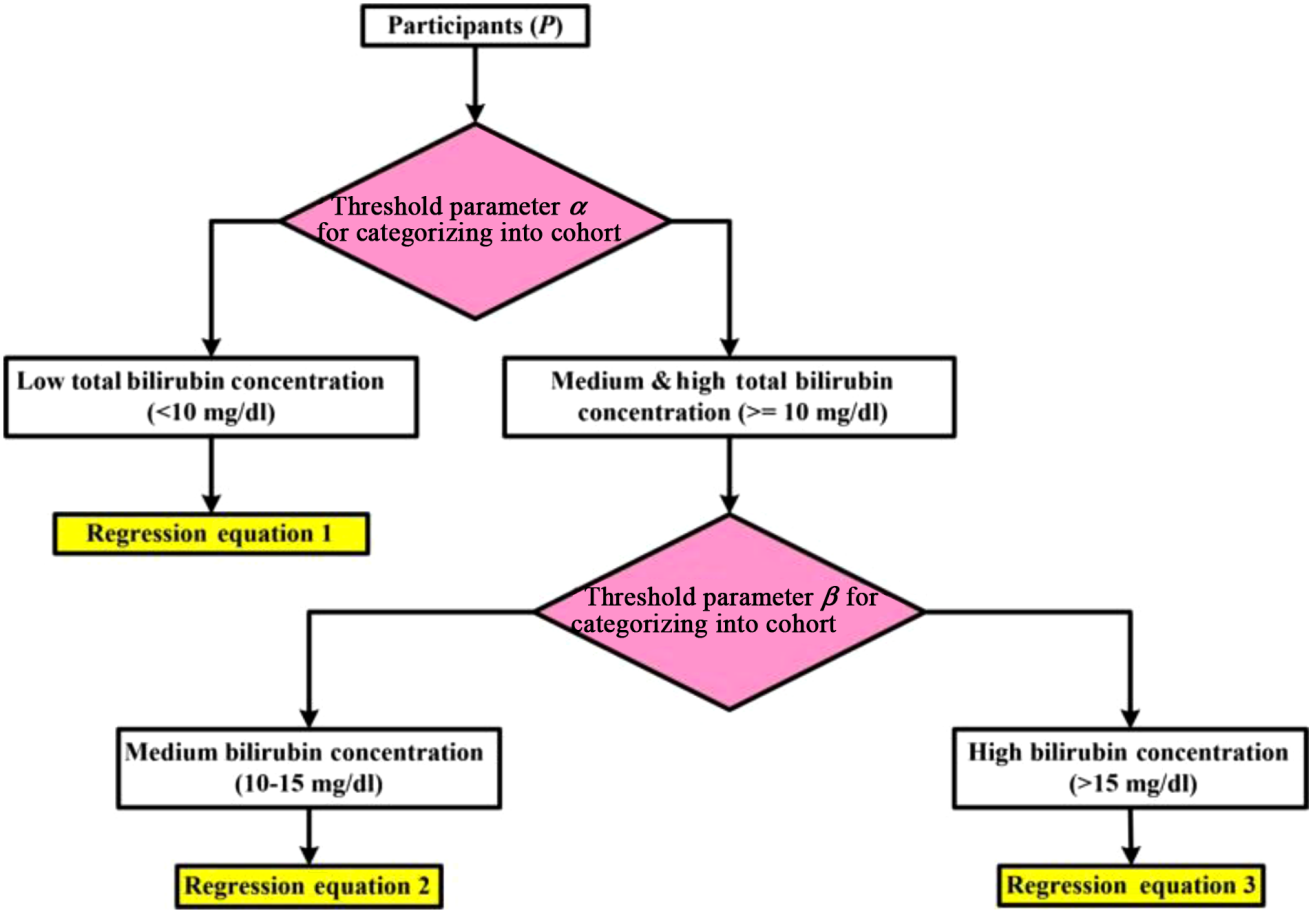
Proposed hierarchical decision tree model.

The measurements were obtained from the laboratory; and the colorimetric method^17^ was used to determine the total and the direct bilirubin concentrations. The 10 to 15  mg/dl range was very common, and most subjects fell into this category. Because it was not a uniform distribution of patients for different ranges of bilirubin concentrations, a hierarchical decision tree model was constructed; it is presented with its associated steps in Fig. 3. Also, a real-time, portable device can easily adapt to this kind of approach in clinical settings to replace the cumbersome spectrometric laboratory determination of total bilirubin in the whole blood of neonates.

#### 2.3.1 Selecting the reference wavelength for all subjects

The subjects were divided into three groups based on the total bilirubin concentrations: (i) low (<10  mg/dl), (ii) medium (10 to 15  mg/dl), and (iii) high (>15  mg/dl). The preprocessed signal obtained as discussed in Sec. 2.2 is plotted as normalized intensity against wavelength for each group in every set. The absorption spectra were recorded at 0 and 15 min time points. We analyzed them for the region of interest (ROI) with no change in intensity with time. This ROI is chosen such that the reference wavelength is the median wavelength of the 10 nm interval, the full-width at half maximum (FWHM) of light-emitting diodes (LEDs).^18^ The normalized intensity of the preprocessed signal is averaged within this band of 10 nm. The step mentioned above uses FWHM to use LEDs as a light source to implement this algorithm in a clinical device prototype. Hence, this entire wavelength interval is used as the reference wavelength ($\lambda_{\mathrm{ref}}$) for further analysis.

#### 2.3.2 Spectral features pertinent to two cohorts viz. <10  mg/dl and ≥10  mg/dl

Neonatal hyperbilirubinemia is frequently monitored after categorizing the neonates based on the levels of severity. In general, the cases with a total bilirubin content of <10  mg/dl are considered mild compared with the neonates with ≥10  mg/dl.^19^ This is why 10  mg/dl total bilirubin concentration was chosen as the first threshold, and the neonates were segregated into two cohorts based on the reports received from the laboratory. For a particular subject, the intensity value at $\lambda_{\mathrm{ref}}$ obtained in the previous step is used to normalize the intensities of all other wavelengths. A spectral measure that depends on the slope between the readings $X_{1}$ nm and $X_{2}$ nm is used to distinguish the two cohorts. Based on this, a “threshold parameter (α)” is formulated that effectively discriminates the neonates with <10 and ≥10  mg/dl total bilirubin concentrations.

#### 2.3.3 Spectral features pertinent to two cohorts viz. 10 to 15 mg/dl and >15 mg/dl

The cases with total bilirubin content >10  mg/dl are further categorized into neonates with 10 to 15  mg/dl and >15  mg/dl total bilirubin concentration. Hence, 15  mg/dl total bilirubin concentration, being the median of the next concentration interval, was chosen as the second threshold to categorize the neonates based on the reports received from the laboratory. The intensities of all other wavelengths were normalized at $\lambda_{\mathrm{ref}}$. A threshold parameter associated with the slope between the intensity values at $X_{1^{\prime }}$ nm and $X_{2^{\prime }}$ nm is used to distinguish the two categories. Hence, a second “threshold parameter (β)” distinguishes the neonates with 10 to 15 mg/dl and >15  mg/dl total bilirubin concentrations.

#### 2.3.4 Correlation prediction

We analyzed the 400 to 500 nm wavelength range to find the region that correlates with the bilirubin absorption for low (<10  mg/dl), medium (10 to 15  mg/dl), and high (>15  mg/dl) total bilirubin concentrations. We carefully observed the raw absorption spectrum of each neonate in all three cohorts and zeroed in on the wavelength that depicted its correlation with the total bilirubin concentration reported from the laboratory. A regression model was constructed from the data points for each of the three cohorts.

#### 2.3.5 Testing the model on 20 unknown samples

The model’s reliability was assessed by following the steps mentioned above on 20 samples with bilirubin concentrations that were not known beforehand.

The sensitivity and specificity of each “threshold parameter” test were calculated as percentages from the numbers of neonates representing each state in the following equation:^20^

Sensitivity=[TP/(TP+FN)]×100, (2)

Specificity=[TN/(FP+TN)]×100, (3)

where TP represents true positives, FN represents false negatives, TN represents true negatives, and FP represents false positives.

## 3 Results and Discussion

**Fig. 4:**
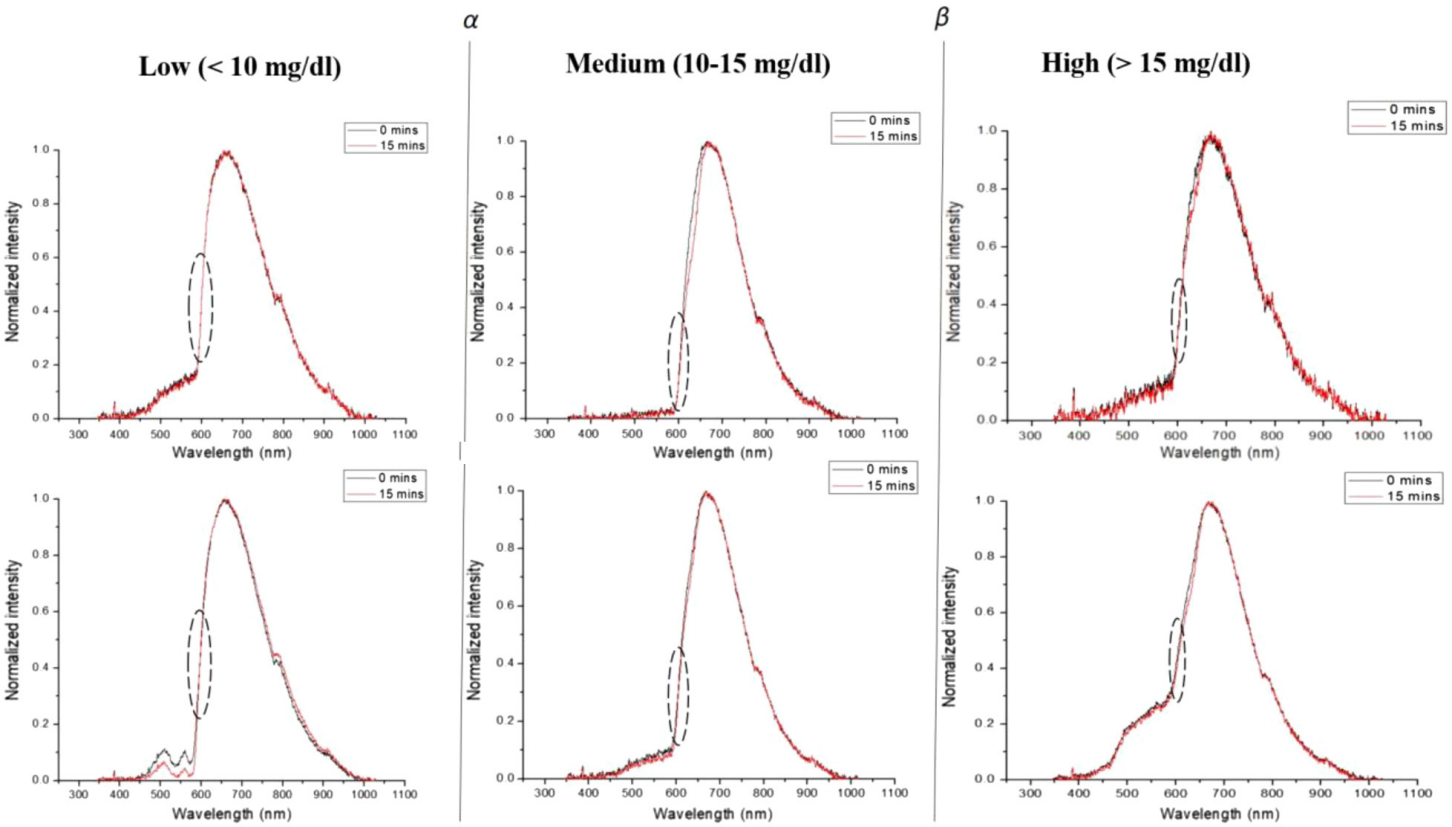
Normalized intensity plotted against wavelength for all three groups viz. low, medium, and high total bilirubin concentrations. At the median wavelength of 605 nm, the graphs exactly overlapped at 0 and 15 min timepoints; hence it was chosen as a reference ($\lambda_{\mathrm{ref}}$).

The reference wavelength was chosen as 605 nm ($\lambda_{\mathrm{ref}}$) because the graphs overlap exactly at 0- and 15-min time points in this wavelength interval, implying little participation in photodegradation within the wavelength range; otherwise, they can be discriminated at all other wavelength regions. Figure 4 shows this feature for all three cohorts: the low, medium, and high groups in which the neonates are divided.

**Fig. 5:**
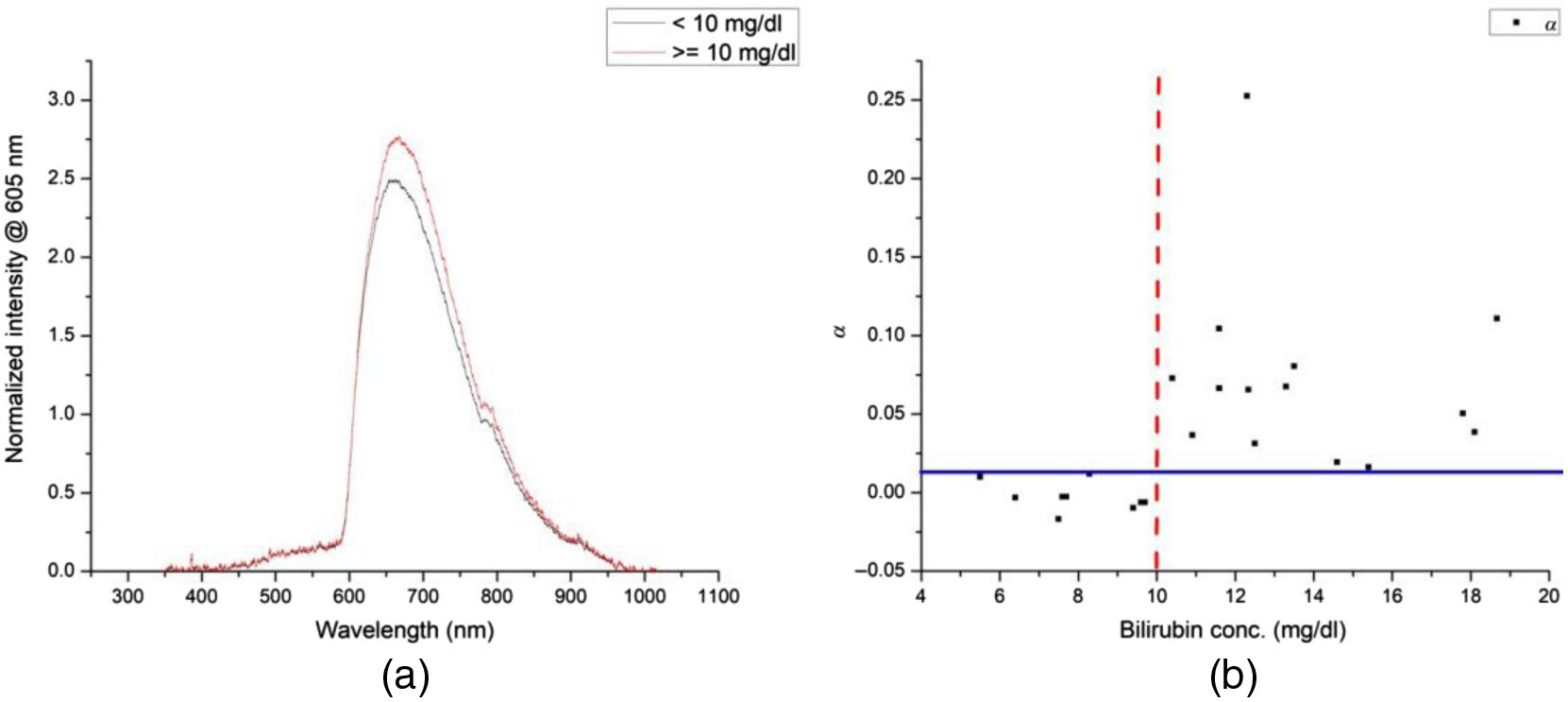
(a) Comparison of normalized intensity at 605 nm ($\lambda_{\mathrm{ref}}$) between the two cohorts viz. <10  mg/dl and ≥10  mg/dl. (b) Distribution of parameter α over the known bilirubin concentrations. The vertical red dashed line marks the boundary for 10  mg/dl, and the horizontal blue line marks the α value that distinguishes the two cohorts.

The neonates were divided into two cohorts. Ten neonates belonged to <10  mg/dl bilirubin concentration, and 20 neonates belonged to the ≥10  mg/dl bilirubin concentration based on the information provided by the laboratory. The preprocessed signal was normalized with 605 nm ($\lambda_{\mathrm{ref}}$) as the reference median wavelength for each neonate. This normalized intensity was further averaged over wavelengths for all subjects in the two cohorts and compared to identify the pertinent spectral features. Figure 5(a) compares the two cohorts; it is observed that, at the median wavelength of 660 nm, there is a perturbation from the normal Gaussian curve that can be exploited as a quantitative difference between the two cohorts. This perturbation occurs after the equilibrium is reached in the diazo coupling reaction between free bilirubin isomers and the albumin proteins in the whole blood. In blood, the perturbed spectrums result from the remaining free bilirubin and hemoglobin, also an albumin protein.^21^

Therefore, we deciphered a criterion based on the differential slope assuming 660 nm as the middle wavelength. The slopes of the graph before 660 nm that is at 645 nm and after 660 nm that is at 675 nm are compared for a “threshold parameter α” for each neonate, which is defined as

$$\alpha =\frac{\left(I[675]-I[645]\right)}{I[660]},$$ (4)

where I[λ] represents the normalized intensity at that particular wavelength in λ nm units. Figure 5(b) shows the effectiveness of this parameter to discriminate between the neonates with <10  mg/dl and ≥10  mg/dl total bilirubin concentrations.

It is clearly visible from Fig. 5(b) that α equal to ∼0.0125 can be used as a threshold to discriminate the two types of pathological conditions of neonatal hyperbilirubinemia. This relation may also be attributed to bilirubin being formed as a breakdown product of heme and the absorption of hemoglobin in blood between 620 and 660 nm generally.^22^^,^^23^ In this way, we can initially determine the phototherapy duration and sessions for a neonate based on the α value of the spectra being less than or greater than 0.0125. The specificity of the α test was 90.9%, and the sensitivity was 85%.

**Fig. 6:**
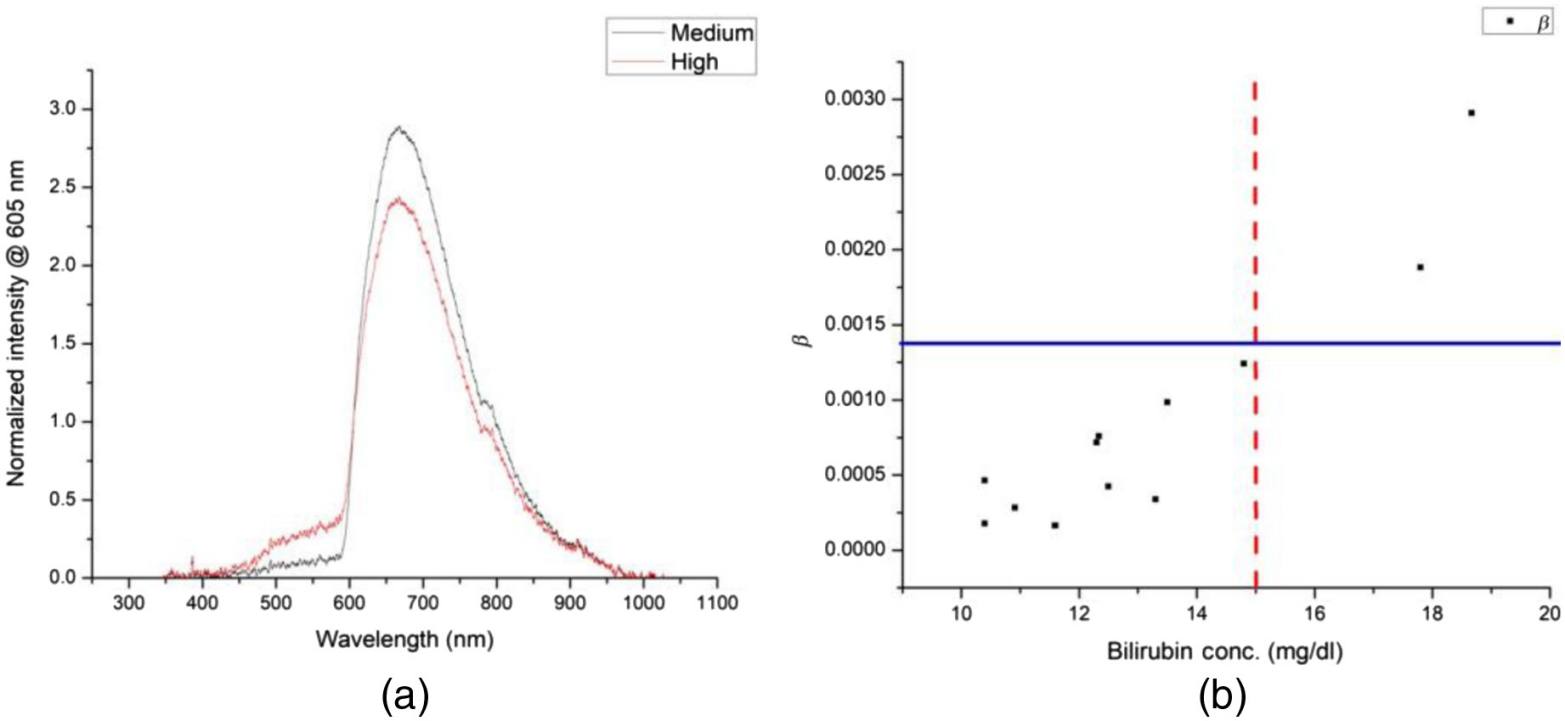
(a) Comparison of normalized intensity at 605 nm ($\lambda_{\mathrm{ref}}$) between the two cohorts viz. medium (10 to 15  mg/dl) and high (>15  mg/dl). (b) Distribution of parameter β over the known bilirubin concentrations. The vertical red dashed line marks the boundary for 15  mg/dl, and the horizontal blue line marks the β value that distinguishes the two cohorts.

We divided the cohort with ≥10  mg/dl total bilirubin concentration into two subsequent groups of 10 to 15 mg/dl and <15  mg/dl. On comparing their absorption spectra, we found that they can be discriminated between 500 and 560 nm, as shown in Fig. 6(a). We estimated the second threshold parameter β as the slope in this wavelength range to distinguish the neonates in the two cohorts in Fig. 6(b):

$$\beta =\frac{I[560]-I[500]}{60},$$ (5)

where I[λ] represents the normalized intensity at that particular wavelength in λ nm units.

The β threshold value observed in Fig. 6(b) is ∼0.0014, which delineates the medium and high total bilirubin concentration cohorts. Methemoglobin peaks between 500 and 550 nm in whole blood^24^ and is formed by the irreversible oxidation of hemoglobin. The specificity of the β test was 91.6%, and the sensitivity was 100%. It is imperative to analyze the concentration of methemoglobin for accurate determination of bilirubin as the absorption spectra of hemoglobin derivatives are conjectured to interfere with that of neonatal bilirubin in whole blood.^21^^,^^25^

**Fig. 7:**
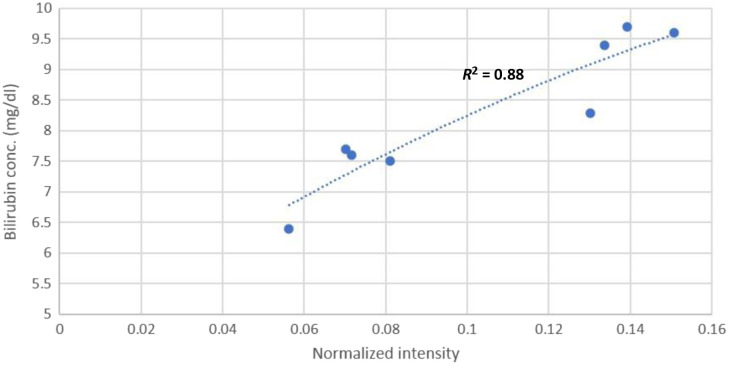
Intensity data points at 492±5  nm are plotted against their respective total bilirubin concentrations of the patients belonging to the low cohort. The polynomial (order = 2) regression plot yields $R^{2}=0.88$.

After segregating the patients into their respective cohorts of low, medium, and high total bilirubin concentrations, we carefully analyze the bilirubin absorption spectrum in the wavelength range of 400 to 500 nm for each cohort. It is required to accurately predict the total bilirubin concentration based on its correlation with a particular wavelength in a cohort. As shown in Fig. 7, we found that 492±5  nm correlates well with the patients in the cohort with low total bilirubin concentration.

With $R^{2}=0.88$, the regression plot yielded the following polynomial (order = 2) equation:

$$y=-78.019x^{2}+45.675x+4.4615.$$ (6)

**Fig. 8:**
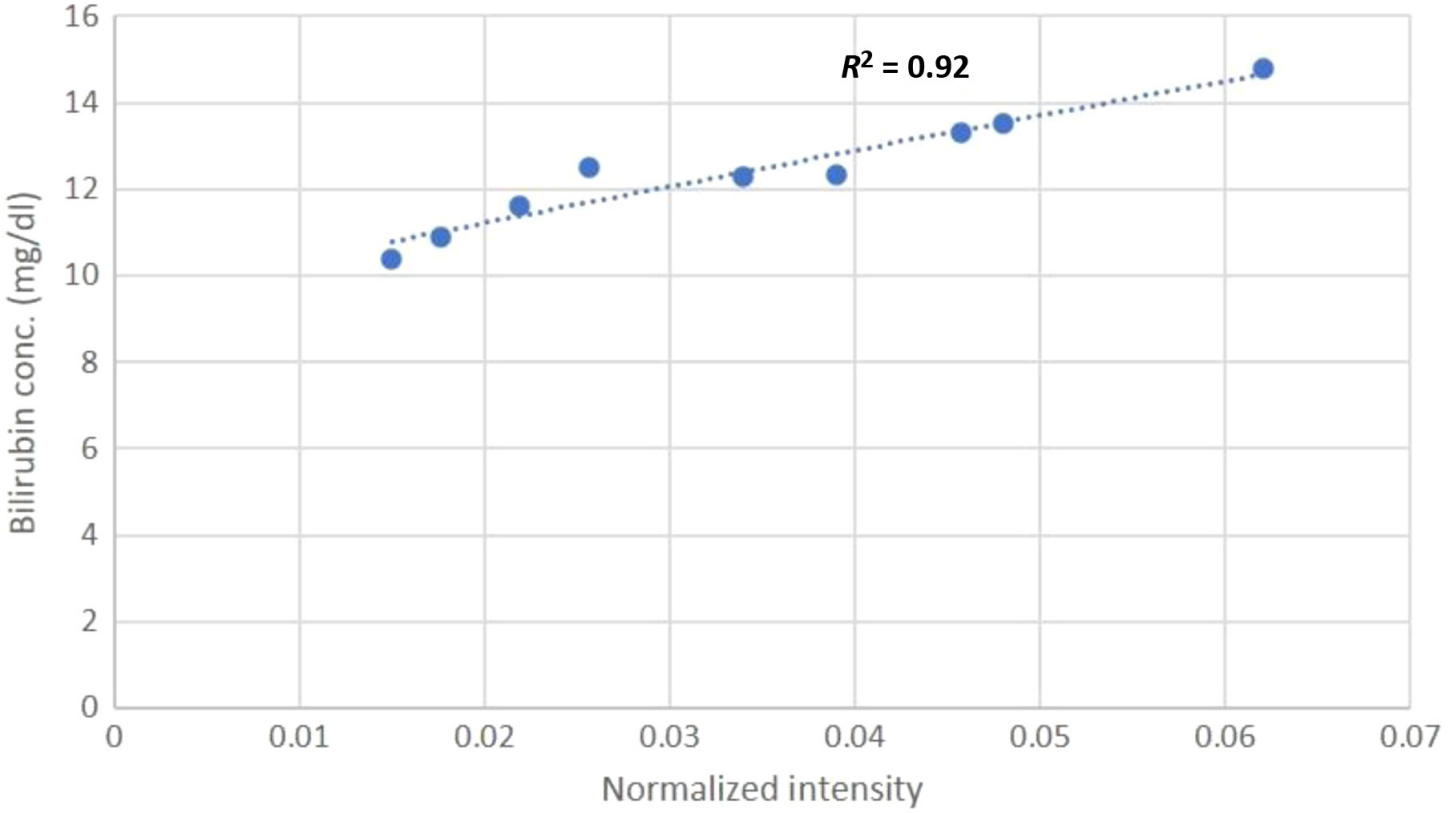
Intensity data points at 468±5  nm are plotted against their respective total bilirubin concentrations of the patients belonging to the low cohort. The polynomial (order = 2) regression plot yields $R^{2}=0.92$.

**Fig. 9:**
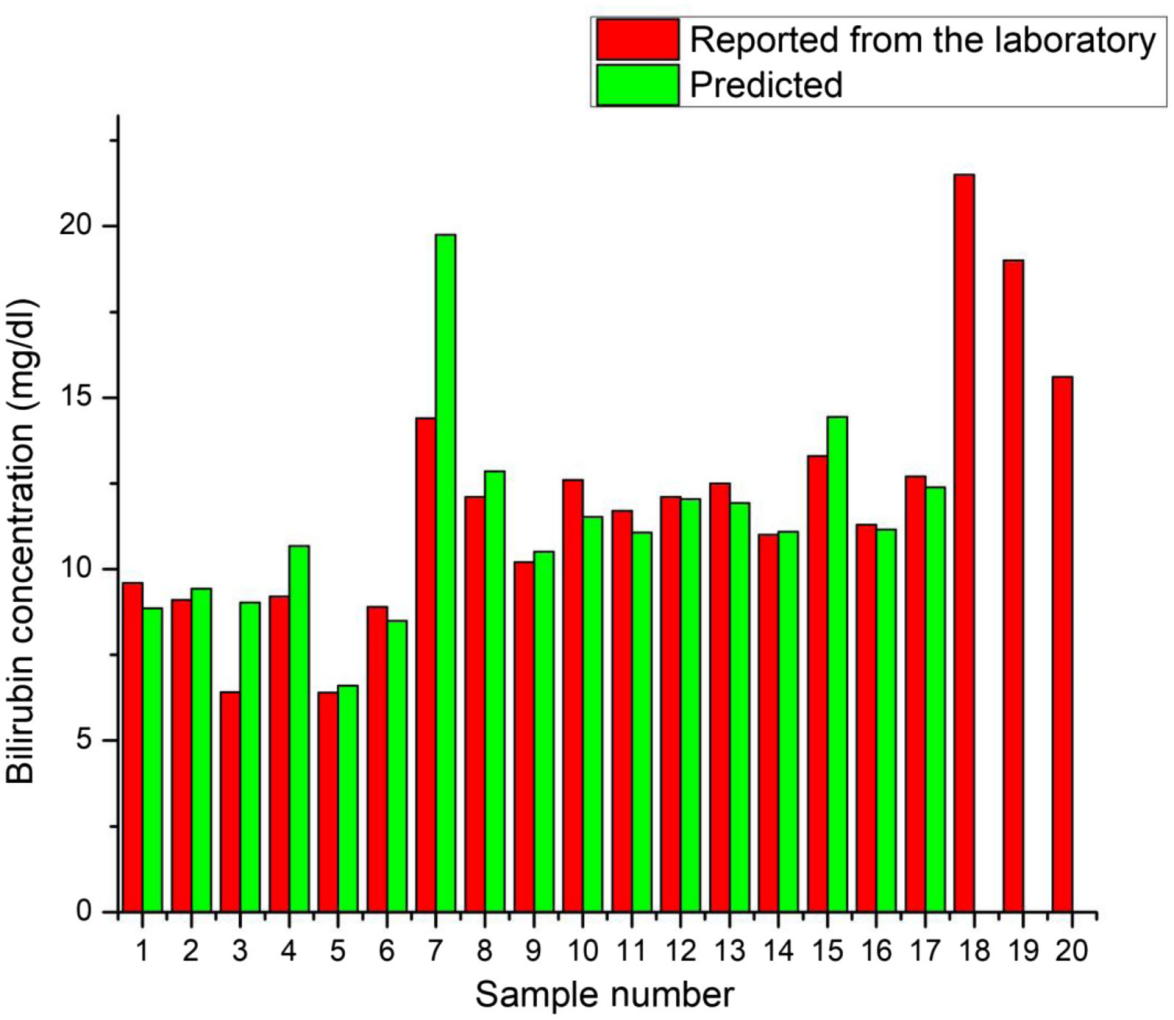
Bar graph represents the bilirubin concentration data reported from the laboratory and predicted with our proposed model for each sample.

On a similar note, the polynomial (order = 2) regression model yields the best results for the medium (10 to 15  mg/dl) total bilirubin concentration at 468±5  nm; the curve fitting yields the following correlation equation with $R^{2}=0.92$ as shown in Fig. 8:

$$y=-79.709x^{2}+88.443x+7.4801,$$ (7)

where x is the average normalized intensity of the sample and y is the total bilirubin concentration in whole blood in Eqs. (6) and (7). The model was tested on 20 samples of the testing group, and the results are shown in Table 1 and Fig. 9.

**Table 1:** Values obtained for the unknown samples from our proposed model.

Sl. no.	Bilirubin conc. (in mg/dl)	α	β	Normalized intensity @ 492 ± 5 nm	Normalized intensity @ 468 ± 5 nm	Predicted bilirubin conc. (in mg/dl)
1	9.6	−0.0476	—	0.12161	—	8.86
2	9.1	−3.6952E-4	—	0.14443	—	9.43
3	**6.41**	**0.04074**	—	**0.12773**	—	**9.02**
4	**9.2**	**0.00209**	—	**0.21509**	—	**10.67**
5	6.4	−0.02099	—	0.05157	—	6.61
6	8.9	−0.06989	—	0.10838	—	8.49
7	**14.4**	**−0.01421**	**0.00322**	**—**	**0.13154**	**19.75**
8	12.1	0.04826	7.30125E-4	—	0.03963	12.86
9	10.2	0.02115	8.66687E-4	—	0.01175	10.51
10	12.6	0.01246	6.00172E-4	—	0.02351	11.52
11	11.7	0.06474	4.30757E-4	—	0.01832	11.07
12	12.1	0.05962	1.91689E-4	—	0.02969	12.04
13	12.5	0.04656	2.7896E-4	—	0.02845	11.93
14	11	0.08559	2.7938E-4	—	0.0185	11.09
15	13.3	0.01692	1.39385E-4	—	0.05925	14.44
16	11.3	0.10341	1.81991E-4	—	0.01929	11.16
17	12.7	0.01455	6.95115E-4	—	0.0338	12.38
18	21.5	0.05585	0.0014	—	—	—
19	19	0.23111	0.00214	—	—	—
20	15.6	0.028	0.00135	—	—	—

Note: Bold values correspond to the predicted bilirubin concentrations that deviated significantly from those reported from the laboratory.

We can conclude from Table 1 that the “α” threshold parameter test is valid for 17 out of 20 samples and accurately divides the samples into two cohorts of <10  mg/dl (low) and ≥10  mg/dl total bilirubin concentration. Further, the “β” test applies to 13 out of 14 cases in which it divided the ≥10  mg/dl cohort into medium (10 to 15  mg/dl) and high (>15  mg/dl) groups. Sample numbers 18, 19, and 20 belonged to the group with a high total bilirubin concentration. Because no model could be formulated due to the lack of participation in this group, we correctly predicted 14 out of 17 samples.

The light from the white light source first traverses the glass wall, then enters the whole blood sample, again enters the glass wall after traveling across the width of the capillary, and finally is completely captured by the optical fiber of the spectrometer for detection. The refractive index of a medical grade glass microcapillary is ∼1.5^26^ and that of whole blood varies from 1.35 to 1.36^27^ in the visible range. The absorption satisfies the well-known Beer–Lambert law:

$$I_{\text{out}}=I_{\text{in}}e^{-\alpha_{\mathrm{abs}}L},$$ (8)

$$\alpha_{\mathrm{abs}}\equiv \alpha_{\mathrm{abs}}(\omega ).$$ (9)

The extinction coefficient “$\alpha_{\mathrm{abs}}$” captures both absorption and scattering losses. Given the volume of the blood sample, the dominant contribution comes from absorption. The optical pathlength “L” remains common for all samples. We are, however, interested only in the spectral changes arising from selective absorption, i.e., α≡α(ω). The scattering contributes weakly, and the use of miniscule volume of blood excludes—to a large extent—the highly scattered paths and thus are not major contributors to the extinction coefficient.

However, the optical path itself plays little role, as we focus on the spectral changes that arise due to the selective absorption provided by the blood sample. There are no spectral changes due to the empty glass capillary, and this has been verified. Furthermore, the optical pathlength remains constant for all samples as the capillary setup remains unchanged throughout the study. A fresh glass microcapillary of the same dimensions and make is loaded with the whole blood sample of next subject.

In addition, two major blood chromophores in the human whole blood, bilirubin and hemoglobin, significantly absorb in the visible range. The absorption peaks are 416.57±1.90, 542.71±1.80, and 578.57±1.81  nm due to the erythrocytes and lymphocytes in whole blood.^28^^,^^29^ The first corresponds to bilirubin, which absorbs at the 400 to 500 nm wavelengths for neonates;^30^ the other two peaks correspond to oxygenated hemoglobin, with deoxyhemoglobin absorbing significantly between 640 and 670 nm.^31^ These are generic characteristic of the absorption peaks of whole blood in the visible range. The variation of these absorption peaks arises from whether the blood is arterial or venous and the bilirubin concentration. A more systematic analysis has the potential to reveal other important biomarkers, such as hemoglobin levels, in an appropriately chosen spectral domain.

When light passes through the whole blood sample, the intensity in the blue region (400 to 480 nm) is the least, gradually increasing in the green region (540 to 600 nm) and sharply peaking in the red region (630 to 670 nm). The blue region corresponds to the absorbance by various bilirubin isomers; the green region corresponds to the oxidized hemoglobin, which is both oxyhemoglobin and methemoglobin; and the native hemoglobin belongs to the red region. After a few minutes, the graph oscillates about its mean position on acquiring the absorbance spectrum at 0 min. A conspicuous decrease is observed after 15 min, which is recorded for most samples. The native hemoglobin is an albumin protein that perturbs the conjugation of free bilirubin to albumin proteins in the whole blood.

In its dissolved state, bilirubin behaves as a potent antioxidant, which is why it participates in the biochemical reactions involving oxidized hemoglobin. Therefore, a multispectral absorbance measurement is required to measure the bilirubin biomolecule in whole blood accurately. Bilirubin is an efficient oxygen-scavenging biomolecule.^32^^,^^33^ We have shown that the decrease in the bilirubin concentration in the presence of white light containing 400 to 500 nm^14^ wavelengths shows a decrease in the measured intensity. As this biomolecule starts to deplete or photoisomerize to other forms, the oxygen species in the blood tends to increase, thereby initiating a change in the dynamics between oxygenated hemoglobin/methemoglobin and deoxyhemoglobin, which is why we observe a see–saw movement in the spectrum as discussed in this paper. These changes occur at about 600 to 605 nm as it is equidistant between oxygenated and deoxygenated hemoglobin peaks. We have not recorded the hematocrit values in this study, and we believe that they could be used to refine our model further. The competition between bilirubin photoalteration and hematocrit is well known.^34^ As we carry out our spectral measurements prior to administration of phototherapy with the collected blood sample being protected from ambient light, this photoalteration competition between hematocrit and bilirubin may not be a contributor to our measurements.

Furthermore, we may not need the hematocrit values of neonatal blood; rather, we may require the hemoglobin concentration of the subject. However, the biology is complex, and it cannot be a one-to-one correspondence; therefore, we tackled this problem through a hierarchical multispectral approach as each biomolecule is connected and is affecting the other. Hence, changes in the bilirubin peaks point to changes in the hemoglobin states and concentration. A pathological hemoglobin value may not be required for the subject until he/she suffers from a blood-related disorder and deviates from the commonly acquired spectrum. For this reason, as we mentioned, we have not determined bilirubin concentrations for outliers or the abnormally high group. Our study currently correlates with only 80% to 85% of cases with low and medium bilirubin levels. The lack of high bilirubin concentration samples needs to be tackled; this is a lacuna in the current work and may be related to hematocrit content.

The slopes of alpha and beta and the regression slopes may further vary as we increase the number of samples in the experiment, but the characteristic wavelength at which they are obtained corresponds to various chromophores in blood and would remain the same within a bandwidth of ±5  nm. For this reason, as we mentioned, this is a preliminary study to determine bilirubin levels in whole blood without needing any pre-treatment in real time with only a few microliters of the whole blood sample. We show that a hierarchical multispectral biostatistical model can potentially solve the problem of accurately determining bilirubin blood levels in neonatal jaundice. However, we need a larger study from different population cohorts to clinically translate this concept into a point-of-care product, which is a limitation of the current study.

The slopes in the spectral domain were used in contrast to absolute values of absorption as absolute values can be misleading. However, the relative molecular contributions are well captured by the differential absorption in the spectral domain, and this spectrally diverse information was used in the study. The differential information will remain independent of the specific implementation involving a varied choice of sources/detectors and geometry of the configurations. The statistical analysis is standard and would certainly improve with a larger study.

As the experiments were carried out on clinical samples, once the subject is diagnosed with neonatal hyperbilirubinemia/jaundice, he/she is kept in a phototherapy unit in the clinic. The blood is drawn at regular hourly intervals to determine bilirubin levels, including prior to the administration of phototherapy. Hence, the experiment cannot be repeated for the same clinical sample multiple times. Therefore, as mentioned, the p-value cannot be provided for this experimental study. However, a larger study is warranted, and our work provides definite directions for such a study.

## 4 Conclusions and Future Scope

In this paper, we have proposed a self-referenced label-free approach for the real-time determination of total bilirubin in the whole blood of neonates. We decomposed the problem into three parts. We first determined if the total bilirubin concentration of the neonate was <10  mg/dl or ≥10  mg/dl, with the threshold of the α parameter being 0.0125 for the two cohorts. Then, the ≥10  mg/dl was categorized into medium (10 to 15  mg/dl) and high (>15  mg/dl) groups using the β threshold parameter that was estimated to be 0.0014. The total bilirubin concentration was correlated with the average normalized intensity between 400 and 500 nm in the diseased newborns, and the regression equations that correlated with particular wavelengths in each cohort were found. The reliability model was then tested on 20 newborns for whom we did not know the total bilirubin concentration initially. The results revealed that the model could be used to diagnose neonatal hyperbilirubinemia. The total bilirubin concentration can be predicted in whole blood in a short duration of time with accuracy using only a few μl of whole blood samples.

We could not test the model on samples with total bilirubin values >15  mg/dl in whole blood because of insufficient participation in that group. Also, the number of samples could be increased in both the training and test groups to tune further the parameters and formulate a more robust model along the same lines as described above to assess the model’s predictability. We also found that the samples with serum that was separated from the blood within a few minutes of light exposure did not adhere to the model and were labeled outliers. The inclusion of more participants in the study will standardize the results and make the model more concrete.

We have devised a statistical approach based on absorption measurements at a few wavelengths that can be adapted clinically to automatically determine total bilirubin in the whole blood, particularly for newborns suffering from neonatal hyperbilirubinemia. An ordinary glass microcapillary where the sample was loaded used only about 3 to 5  μl of whole blood. Therefore, it eliminates the requirement of using a few milliliters of blood drawn via syringe needles bypassing the cumbersome, time-taking spectrometric method. The finite bandwidth LEDs can be chosen as portable light sources with a median wavelength as their dominant wavelength. A photodetector located centrally in front of the microcapillary sequentially records signals at the wavelengths mentioned above to provide an automated, real-time, portable, and cost-effective yet reliable clinical bilirubinometer.
